# Strangulated Inguinal Hernia in Infants with Report of a Case

**Published:** 1908-01

**Authors:** John Egerton Cannaday

**Affiliations:** Hansford, West Virginia, Surgeon-In-Charge, Sheltering Arms Hospital


### STRANGULATED INGUINAL HERNIA IN INFANTS WITH REPORT OF A CASE.

---

By John Egerton Cannaday, M. D., Hansford, W. Va.,

Surgeon-In-Charge, Sheltering Arms Hospital.

---

As compared with its frequency in adults, strangulated hernia is unusual in infants, the laxity of tissues combined with the absence of many of the usual exciting causes, tending to prevent it. So rare is it that not one half of one per cent of the cases of hernia occuring in infants are strangulated. However, when it does occur, the lack of resistance in the subject renders the indications for surgical relief even more insistent than when this accident happens to the adult. Continental surgeons, especially those of England and Scotland, operate rather freely on hernia occuring in infants. In this country the persistent use of a teuss for several years is thought well of. If that fails to effect a cure, then the radical operation should be resorted to. The infant, like the aged and infirm, is in many particulars an unsatisfactory subject for surgical intervention. In accordance with the rule of frequency which obtains with adults, about three males are afflicted to one female. The abnormally large inguinal opening in the infant is usually of congenital origin, unlike the acquired condition of the aged.

A nearby loop of the intestine becomes crowded into the sac; with the lessening of the pressure which forced the bowel down, the dilated hernial ring contracts and grasps the bowel; the imprisoned intestine is irreducible and cannot free itself; there is a stasis of the fecal current and interference with the circulation (producing, in mild cases, venous congestion, and in severe cases, absolute cessation of circulation.) The ultimate result of strangulation is gangrene, the rapidity of development varying with the conditions present. The vessel anastomoses are few, the fecal content decomposes easily, and bacterial invasion of the sac is rapid. The intestine becomes friable; gangrene begins in small areas where the circulation is poorest; these discrete patches later become confluent. Considerable disturbance takes place in the loop leading to the hernia; putrefaction of the stagnant contents occurs with the resultant gas distension; reversed peristalsis and other disturbances along with peritonitis. Distension may be so great as to obliterate the liver and splenic dulness.

In the symptomatology of strangulated hernia, we have three symptom groups, arranged from the point of time in the order named: Strangulation shock; intestinal obstruction and sepsis. Strangulation shock is as distinct an entity as that produces by torsion or compression of the testicle. The symptoms comprise, nausea, vomiting, small thready pulse, pallor of the skin and mucous membranes, cold perspiration and other disturbances of the general condition. The intestinal obstruction produces meteorism varying in accordance with the high or low constriction of the gut, the vomitus becomes partly or wholly fecal in character. The higher the obstruction, the earlier the vomiting comes on. Sepsis brings about restlessness, lack of appetite, nausea, peritonitis of gradual or sudden onset; a general intoxication from the gangrenous or suppurative condition of the sac may intervene. When peritonitis develops suddenly from perforation or some other pathological accident, the pain is violent and localized in the beginning, later spreading over the entire abdomen. The symptoms common to continued and increasing sepsis and intestinal obstruction continue and grow worse. Obliguria and even anuria may come on as a result of the diminished amount of water in the body.

The diagnosis is based on the history and the symptoms. The hernia usually feels firmer than before and is often sensitive to the touch. An irreducible mass is present which extends into the abdominal cavity. It may have a certain amount of lateral motion, but up and down motion will be very limited. At times, even when the vitality of the gut is endangered, the symptoms may be so atypical and slight as to make the diagnosis one of doubt. On the other hand, the strangulation of a small omental hernia may occasion extreme symptoms. At the moment of strangulation there is a sharp pain and the hernial mass is increased in size. An accurate history is of much importance in making the diagnosis. There is often a bowel movement from the portion below the hernia, soon after the obstruction takes place. After the strangulation, coughing and straining do not influence the tension.

When left to itself, the prognosis is so grave that recovery would constitute a surgical curiosity. At times, strangulation shock alone is of sufficient severity to kill the patient; pneumonia, inanition, prolonged suppuration or other inter-current trouble may cause a fatal ending.

The treatment in most cases is undoubtedly and unquestionably surgical, so long as the patient is not moribund. When the patient is seen early after the symptoms of strangulation have made their appearance, and when the onset has not been acute, taxis should be resorted to, and in the event of failure, the hernial sac should be opened at once. Taxis is thought by many to be more especially indicated in the very young who are hard to keep clean, and in the aged and infirm, who do not bear operation well. Taxis may waste valuable time, and may bring about the reduction of hernia en bloc or bowel that has lost its vitality. Violent manipulations may cause tearing of the intestine. Before resorting to taxis, the stomach and lower bowel should be irrigated and emptied, the urine should be drawn, muscular relaxation obtained by appropriate posturing and the use of an anaesthetic, and the hernia should be elevated so as to be the highest part of the body. The fluid contained in the sac when opened is an excellent indicator of the condition of the intestine. If the fluid is ill smelling or contains flakes of lymph, the bowel is very likely to be in a bad condition. In attempting to make the decision as to whether a piece of gut is viable or not, the sense of touch may give more information than that of sight. The lifeless gut is flaccid and has the feel of moist blotting paper. Gray brown spots are indicative of gangrene. Resection, and even the formation of an artificial anus will often have to be resorted to. When there has been much infection, the radical operation should be omitted and the wound packed with gauze. In case of doubt, sutures can be placed and left loose to be tied later. When the radical operation is resorted to it is usually best to make use of the more simple and time saving methods. I do not believe that the transplantation of the cord has any particular merit to commend it. Connell and Ferguson are quite certain that it predisposes to epididymitis or other inflammatory lesions. Operation infection should be very rare when a careful rubber glove technic is followed. After ligating the neck of the sac, I often carry the ends of the suture upward between the parietal peritoneum and rectus muscle, and secure the neck of the sac in a higher position so as to obliterate the former funnel-like depression existing at the point of occurrence of the hernia. After dealing with the sac, the hernial opening is closed by the introduction of sutures of thirty day chromic catgut. The idea suggested by Czerny, and later elaborated by Ochsner, that if the ring is denuded of its lining before being closed, union will be assured and recurrences rare, should be one of the basic principles of surgery of this part of the body. The cord is not moved from its normal position, and the superficial is sutured over it with a running suture of plain catgut. The edges of the skin wound are approximated with the usual suture of silk or linen thread.

The operation, as we know it, for the radical cure of hernia, was first performed by Czerny in 1877. The mortality of operations for the radical cure of hernia in skilled hands is from ½ of 1 to 1 per cent. The mortality of strangulated cases for many reasons will always be high.

I will report the following case: The patient was a three months old male baby referred by Dr. H. S. Reger of Macdonald, W. Va. The child developed a complete inguinal hernia soon after birth. This was easily reducible at first, but later difficulty was experienced in returning the bowel to the abdominal cavity. The evening before the patient was brought to the hospital, symptoms of strangulation came on. Attempts were made to reduce the tumor by taxis, first without and later with chloroform anaesthesia, but unsuccessfully. As the condition of the child was becoming serious, it was brought to the Sheltering Arms Hospital the next morning and operated on immediately. The child was weak and considerably shocked. For this reason it was deemed advisable to make use of local anaesthesia. A sterile 2 per cent solution of beta-eucaine was used. The customary method of anaesthetizing the nerves supplying the parts involved and also the line of incision was followed. I at once dissected the structures down to the constriction, opened the sac, enlarged the ring, and, as the imprisoned loop of gut seemed viable, returned the strangulated intestine to the abdomen. I then closed the hernial ring with chromicized catgut. During the course of the operation, the parts were handled as gently as possible; but little shock was manifested and the baby slept during the greater part of the time. To prevent wound contamination, a dressing composed of a few fibres of cotton and some flexible collodion was used. The baby rallied promptly and in a few hours was nursing at its mother's breast. The course of convalescence was uneventful and so far, six months afterwards, there had been no return of the hernia.

To summarize, I would note the infrequent but occasional occurence of strangulated hernia in infants. The ease of a reasonable certainty in the diagnosis of this condition. The dangers of taxis by reason of the fragility of the structures involved. The dangers of operations in infants have perhaps been over-estimated. By proper precaution and care, the risks of post operative infection can be largely eliminated. The shock incident to anaesthesia and prolonged manipulation can be avoided by the proper use of a sterile and practically non-toxic anaesthetic and by rapid and careful work.

